# Food Rating Scale in Food Services: From Development to Assessment of a Strategy for Consumer Healthier Choices

**DOI:** 10.3390/nu10091303

**Published:** 2018-09-14

**Authors:** Larissa Mazocco, Rita De Cássia Coelho Almeida Akutsu, Raquel Braz Assunção Botelho, Izabel Cristina Rodrigues Da Silva, Raquel Adjafre, Renata Puppin Zandonadi

**Affiliations:** 1 Research Group in Nutritional and Nourishment Quality, Department of Nutrition, University of Brasilia, Brasilia, DF 70910-900, Brazil; mazoccolarissa@gmail.com (L.M.); rita.akutsu@gmail.com (R.D.C.C.A.A.); raquel.adjafre@gmail.com (R.A.); renatapz@yahoo.com.br (R.P.Z.); 2 Department of Pharmacy, Faculty of Ceilandia, University of Brasilia, Brasilia, DF 70910-900, Brazil; belbiomedica@gmail.com

**Keywords:** nutritional education, energy density, sodium, food choice, rating scale

## Abstract

This study aimed to create an easy tool to identify healthier choices for meal assembly in food services (self-service restaurants) and to allow consumers to compose their plates to make healthier choices. It is an interventional study, and the first step was setting healthy food parameters to design a rating scale. The first evaluation criterion was based on energy density (ED) and sodium content (SC) using “traffic light” color in the dishes’ nameplates; the second criterion was based on food groups; the third criterion was based on ingredients of the meals. After using the classification, we assessed the rating scale in a food service and we evaluated the strategy with its consumers. To evaluate the effect of the nutritional intervention, we developed a multiple-choice-questionnaire with eight questions to measure the impact on consumer food choices quantitatively. The dish nameplate allows identification of healthier choices regarding SC and/or ED by colors; ingredients that compose the dish; the food group and the serving size, helping the identification of the amount of food to compose the meal. Banners helped consumers to understand the information. After four weeks, all the consumers (*n* = 1000) received questionnaires regarding their comprehension of the classification. The questionnaire presented an ICC of 0.71. Most of the preparations (61%) were inadequate based on ED and/or SC at the studied food service. A total of 556 consumers returned questionnaires, and 86.3% of them observed the rating scale as a nutritional strategy. Almost 55% (*n* = 261) of consumers reported changes in food choice after reading the dishes nameplates. The items with greater impact on consumer change in eating behavior were the use of colors as an indicator of nutritional quality, portion size information and ingredients list. Almost 25% of the consumers that changed their eating behavior noticed more than three items presented on the nameplate.

## 1. Introduction

In recent decades, globalization and modernization have been promoting lifestyle, societal and feeding habit changes. It is a great challenge to maintain cultural food habits in the population since the processes of globalization and urbanization have changed the food profile of the community [1]. It is noteworthy that people are eating out and meals prepared at home have been replaced by fast meals, in places of easy access and fast service [2,3]. As part of the modern lifestyle, eating out has a central role in providing a sense of quality in diets and ensuring public health [4]. In this context, eating out has been increasing the consumption of fatty, salty and sugary foods, with a high energy density (ED) and low fiber content [5,6,7], as well as larger portion sizes [4]. Therefore, the analysis of the contribution of eating out to the daily dietary intake has an integral part in nutrition research, due to the rising concern about the role of dietary choices in chronic diseases [2].

In Brazil, a wide type of food service is the kilo buffets, restaurants that serve a varied menu and price relates to the weight of the choices in the plate. Consumers, after choosing their dishes and assembling plates, direct themselves to a weighing scale to pay according to the served amount. This type of service helps consumers to be more careful when portioning because they pay for what is on the plate no matter if there are leftovers. Consumers can assemble a meal according to their preferences among the offered preparations. These dishes are usually established by dietitians or chefs, based on several criteria which include meal cost and time to prepare [4]. We do not choose only by the offered preparations in a restaurant, but also by cultural factors and personal beliefs [2]. A study showed that restaurants that did not provide nutrition information to consumers had higher amounts of calories, fat, and sodium on their menus than restaurants that made nutrition information readily available. To encourage the promotion of healthy menu items, public health practitioners can help to create consumer awareness about sodium in restaurants and to encourage menu changes by reducing sodium [8]. Therefore, it is important to offer and to promote healthier food choices, since poor food choices tend to lead to an overconsumption of energy-dense foods and low intake of fruit and vegetables. An increase in the risk of non-communicable diseases relates to food choices, and some conducted research shows how to achieve healthier consumer food choices [4,9,10].

Although consumers can read and compare Nutrition Fact labels when purchasing packaged foods, acquiring information while eating out can be more challenging. Nutrition fact panels are not mandatory for food services in most countries [11]. In this context, studies have been highlighting the empowering element that nutrition information in a food service setting has in allowing consumers to make healthy choices [12,13] to control the advance of chronic diseases [14]. Excess sodium and lipid consumption is a significant health problem that contributes to hypertension, diabetes, obesity and other chronic illnesses which are leading causes of death worldwide [11,15,16,17]. Although the studies show that nutritional information is the most critical strategy to induce healthier food choices, systematic reviews assessed the influence of menu-labeling on food choices and the studies verified that the only exposition of calorie/nutrients labeling in menus is not sufficient to promote healthier food choices [18,19]. The studies which used the exhibition of the nutrient content of foods showed that the healthier choice depends on the understanding of consumers about the information [2,12,13,20]. Indeed, there is an evident need for better consumer education on appropriate portion sizes to aid adherence to a healthy diet and to avoid some chronic diseases such as obesity, diabetes, hypertension, and others [21].

Another systematic review [22] about changing dietary choices of adults toward healthier choices showed that consumer comprehension strategies of labeling information led to an average increase of 15.3% in healthy nutritional choices. However, it is important to highlight that laboratory situations are used in most of the studies and they are not widely generalizable [22]. The interpretational aids can help consumers to appraise the nutrient contribution of specific foods to the overall diet, leading to the consumption and consequently to the production of healthier products [23].

Some studies have been evaluating traffic light labeling to identify healthier foods due to their quick and easy form of recognition [19,23,24,25]. A study conducted in Germany [23] with 420 participants evaluated whether different formats of food labels helped consumers to differentiate between healthy foods and less healthy foods and whether they are likely to have an impact on consumer food choices. The results from the study indicate that the traffic light system showed the best performance on healthier food choice. Another study also evaluated the food choice regarding traffic light, and the authors identified a strong preference of part of the respondents to avoid a basket of foods containing red lights [24]. Even though some countries use traffic light labeling, to the best of our knowledge, there is no report about a tool that promotes easy and immediate identification of food in food service by ED and Sodium Content (SC). Therefore, this study aims to create and to evaluate an easy tool to identify healthier choices for meal assembly in food services and to allow consumers to compose their plates within their habits.

## 2. Materials and Methods

We conducted an interventional study [26] carried out in three steps. The first step was setting healthy food parameters to design a rating scale for dishes: (i) we based the initial assessment criterion on ED and SC; (ii) the second evaluation criterion on food groups; (iii) and the third evaluation criterion on dietary ingredients. The second step was the application of the rating scale in a food service unit (a self-service by the kilo restaurant) by classifying the dishes offered by the food service. Finally, the third step was the statistical analysis. The University of Brasília Ethics Committee approved the study (CAAE: 1.081.189.2015).

(A) Design of the Rating Scale for Foods

(i) Evaluation criterion based on ED and SC

This classification includes ED and SC evaluation of the dishes and “traffic light” colors based on consumption recommendation (Table 1). To classify dishes using these criteria, first, we had to calculate energy values of each dish and we expressed ED in kcal/g of food. According to the Centers for Disease Control and Prevention [27], they classify preparations as: high energy density (4 to 9 kcal/g), medium energy density (1.5 to 4 kcal/g), low energy density (0.7 to 1.5 kcal/g) and very low energy density (0 to 0.6 kcal/g).

Afterwards, we evaluated sodium (Na) content by the UK Food Standards Agency parameters [28] which classify food by sodium content (mg) in 100 g of food: high content (>600 mg Na), medium content (>120 and ≤600 mg Na) and low content (≤120 mg Na).

With both criteria established (color and SC and ED), we classified all the preparations. Green preparations contained low SC and low ED, and we recommended them freely to consumers. Preparations classified with yellow color should present low SC and medium ED; or medium SC and low ED; or medium SC and ED. For these preparations, we recommended consumption for only 2 or 3 times a week or a little portion size for daily consumption.

We used the red classification for dishes that presented high SC or ED. Since these preparations present high SC or ED, we recommended to reduce the consumption to once a week or to consume a tiny portion when the consumption occurs more than once a week.

We used this color classification to construct the card to identify (nameplate) the food quality. Therefore, we have the information about SC and ED indicated by colors. To classify each preparation in a restaurant based on the references, we created the technical preparation files (TPF) [29] to evaluate the composition of the dishes.

(ii) Evaluation criterion based on food groups

Beyond the food classification by colors according to ED and SC, we separated meal components into food groups to contemplate the consumption of different types of nutrients and to determine the plate to consumer needs regarding portions of each food group. Therefore, we used the Brazilian Food Guide (BFG) [30,31] and “My plate” [32] to share (proportionally) groups of foods that should be consumed at lunch to compose a healthy plate for Brazilians, using total energetic value (TEV) percentage. We adjusted the classifications and recommendations according to Brazilian habits [5,33,34] and lunch recommendations [30] to propose the “Brazilian healthier lunch plate”. Therefore, each group of this classification was named by letters to facilitate its identification on the dish nameplate (Table 1). We used the BFG to adjust the graphical representation of “my plate” food groups to bring Brazilian food habits to the study. We chose My plate due to its easy graphical tool to identify the proportion of components. Therefore, we decided to use a graphical tool as in My plate, but the distribution of the plate related to the percentage of TEV. This strategy was necessary since there are considerable variations in food preparations, such as ingredients, cooking methods and other factors that influence the weight and the size of food portions. Therefore, if the plate were based on weight or portion size, we would provide information that did not correspond to each preparation.

To adjust the percentage and groups to propose the “Brazilian healthier plate for lunch”, we considered the following factors for each group:

(a) We separated grains and beans of the big groups of BFG and “My plate” because, in Brazil, it is common to consume white rice and beans in stock at lunchtime [30,34]. The BFG recommends the use of two parts of rice and 1 part of beans in stock. Therefore, we suggest the maintenance of the Brazilian habit, using healthy grains such as brown rice with beans in stock.
(b) Since “My plate” considers starchy vegetables on the group of vegetables differently from BFG (which includes in grains group), we classified starchy vegetables as in “My plate” classification. We increased the percentage of vegetables in the plate and preserved the Brazilian habit of rice consumption at lunch. In BFG, vegetables and fruits compose 15% of TEV, and we proposed 20% of TEV from vegetables and 10% from fruits since fruits could be consumed as desserts at lunch, as a Brazilian habit. Therefore, we doubled the percentage for these groups, which frequently presented high fiber content and low ED.
(c) Dairy products are rich in protein. BFG recommends 25% of TEV from meats, eggs, and dairy products. “My plate” considers 20% of the plate from protein and recommends the intake of one portion of dairy products. Therefore, we chose to classify these products as a protein group and to apply 25% of TEV, in accordance with BFG recommendations. In Brazil, we consider protein dishes those with higher protein content, which can be composed of meat, dairy products or eggs. Therefore, we included dairy products in the protein group to compose the meal. It is also important to highlight that, although beans present a good source of protein, they are not in the protein group in BFG, and beans are in the grains group. Moreover, since beans are almost mandatory in a Brazilian’s lunch plate due to their habit, we opted to classify beans in a separate group.
(d) In Brazil, it is a habit to consume healthy oils (like olive oil) and/or seeds at lunch. Since these products are healthy when consumed in little portions (because they have high ED) and BFG recommends their consumption, we included them on the “Brazilian healthier plate for lunch” classification to compose the plate. It is important to highlight that the BFG stated a maximum of 15% of TEV from oil, fats, seeds, and added sugar. However, in our recommendation, we did not recommend added sugar.
(e) We maintained the guidance of 800 kcal at lunch, in accordance with BFG recommendations because, in Brazil, we have almost 40% of TEV consumed at lunch.

(iii) Evaluation criterion based on food ingredients

The last evaluation criterion we based on dietary components. Mainly the list of ingredients is important because there are dietary restrictions such as gluten, milk/lactose, nuts, eggs and seafood [35,36,37] as the most prevalent in the population. Since we recommend describing the ingredients in the dish nameplates, consumers should easily identify seafood, eggs and nuts and other allergens.

(B) Application of Rating Scale in a Food Service Unit

(i) Selection of food service

We selected a convenient food service unit (Brasília/Brazil) serving meals in a self-service style paid by the kilo. This type of service buffet is characteristic of Brazil, and it consists of offering dishes in thermal counters, which consumers can choose the dishes that will compose their plates. The responsibility for constructing the menu is based on the choices of the Brazilian food habits offering a great variety of dishes. The self-service where the study occurred offers about 1000 meals per day at lunchtime. The food service is inside a judiciary building of the Brazilian government.

(ii) Technical preparation files

TPF describe preparations on their ingredients and their amounts, the way they are prepared, cooking indicators as well as the nutritional information of the dish [29,38,39]. To list and to evaluate the ingredients and each food composition, we used TPFs [29]. Our group followed all prepared dishes during their production to develop the TPFs. For each dish, we listed all the ingredients used, and we recorded the gross and liquid weights for each ingredient. There were also records of the preparation yields and cooking factor (Fcy), defined as the ratio between the quantity of cooked food (ready to use) and the amount of raw food used in the dish (Fcy = cooked food weight/liquid weight). In the TPF, there was also a detailed description of the preparation steps, cooking method, type of equipment and appliances. We determined portions of each preparation using the appliance used to serve the preparation (the amount of the preparation that fits on the utensil was weighted to define the portion).

Based on the data recorded in the TPFs, we calculated the nutritional value using the information available in the Brazilian Food Composition Table [40]. When this information did not exist on the table, we used scientific publications and labels of food products. Then, data entered into the system database (*DietWin Professional* version 2008^®^).

(iii) Food service application of the food rating scale

To apply the rating scale at the food service, we used nameplates sizing 7.0 × 11.0 cm for the identification of each preparation. We placed cards in front of the preparation, and they expressed the identification criteria (color, food group, serving size, and ingredients).

To explain this nutritional education tool to consumers, we used interpretive boards located near the buffet (banners). The boards contained information to help consumers to understand the new rating scale for food. Additionally, we used another board to stimulate high consumption of vegetables, since they are low ED and SC.

To evaluate the effect of the nutritional intervention, we developed a multiple-choice-questionnaire with eight questions to measure the impact on consumer food choices quantitatively. This questionnaire contains demographic variables questions, the lunch frequency at the food service, intervention perception, comprehension of the educational tools, and change intensity on food choice and intervention items most observed by consumers during research. After four weeks, all the consumers of the food service (*n* = 1000) received this questionnaire regarding their comprehension of the classification through the Internet system of their workstation and returned them electronically without identifying themselves.

(C) Statistical Analyses

We used the Statistical Package for the Social Sciences (SPSS) version 21.0 to conduct the statistical analysis. We calculated frequencies and percentages for categorical variables, and the means and standard deviations for a continuous variable. Chi-square or Fisher’s exact tests were executed for categorical variables and were calculate odds ratios (OR) and 95% confidence intervals (95% CI). Values of *p* < 0.05 were considered statistically significant. For the reliability of the instrument, we used the interclass correlation test (ICC).

## 3. Results

**Figure 1:**
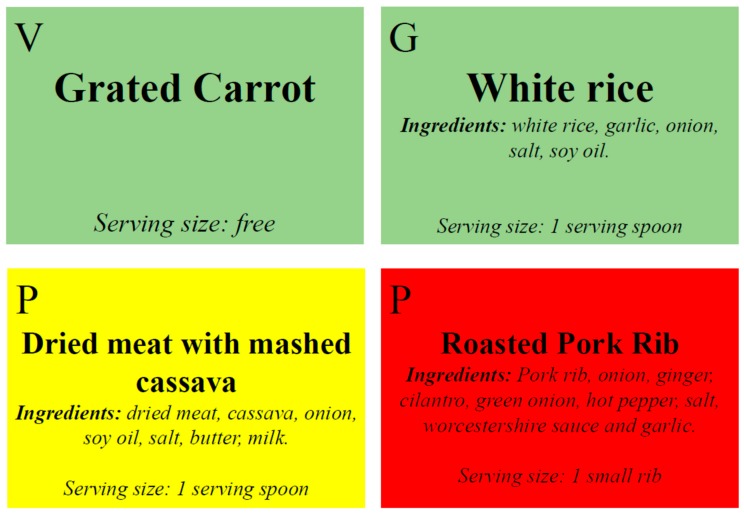
Examples of food nameplates proposed to the restaurant by using the rating scale. V = Vegetable; G = grains; P = Protein.

We constructed dish nameplates that contain the rating scale to help consumers to make healthier plates. Figure 1 presents, as proposed by the rating scale, examples of dish nameplates used at the restaurant.

**Figure 2:**
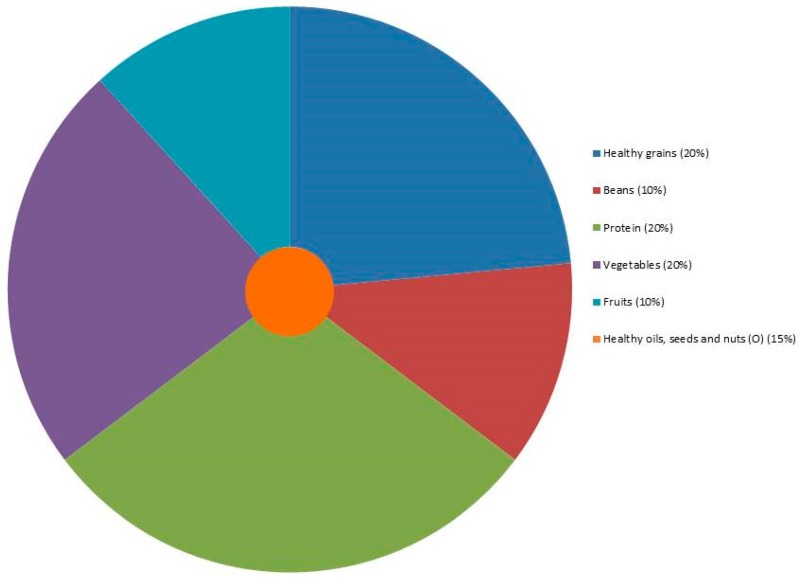
“Brazilian healthier lunch plate” based on a percentage of total energetic value.

The dish nameplates allow identification of healthier choices regarding SC and/or ED by colors; ingredients that compose foods; the food group and serving size. To facilitate the composition of the plate, we proposed a graphic representation of a meal as “Brazilian healthier lunch plate” (BHLP). We based the new distribution and classification of food groups for BHLP (Figure 2) on BFG and “My Plate.” As described by BFG, we used a percentage of TEV for the distribution.

### Food Service Application of the Food Rating Scale

Based on the established parameters in this study for the rating scale to create dish nameplates, we evaluated menu of one food service unit. We constructed and analyzed 144 TPFs from this food service. The ED, SC and mean food portions of each dish are in Table 2. In general, in this food service, 88 (61%) of the TPFs presented high ED and/or high SC. From the 88 TPFs, 40% (*n* = 35) were high regarding only SC; 33% (*n* = 29) only in ED and 27% (*n* = 24) presented both, high ED and SC.

Regarding the classification by colors, in this food service, we mostly classified the dishes as yellow or red, salad sauce (100%), grains (71%), and proteins (65%) (Table 2). The green color nameplate, regarding low ED and SC, classified fruits and raw vegetables.

After classifying all the preparations and creating the dish nameplates, consumers could compose their plates according to SC, ED, food groups and ingredients. Banners helped them to understand the information. After four weeks, all the consumers (*n* = 1000) received questionnaires regarding their comprehension of the classification. The questionnaire presented an ICC of 0.71 showing good repeatability results.

Consumers were free to answer the questionnaire, and we reminded them several times through the Internet system. A total of 556 consumers returned questionnaires (55.6%), and 86.3% (*n* = 480) of them observed the nutritional education strategy adopted by the food service. Consumers were mostly women (53%), up to 35 years old (52%), with a postgraduate degree (53%), married (64%) and without children (50%). The majority (86%) eat at the restaurant 3 to 5 times a week.

The main question of the instrument was to evaluate if consumers changed their food choices. From the 480 consumers that noted the educational strategy, 54.5% (*n* = 261) reported positive changes in food choice (better food choices) during lunch after reading the nameplate cards.

Seventy-six consumers did not notice the nutritional strategy, being 46% female and 54% male; 39% up to 35 years old, 51% married, and 54% with children. About education, this group presented 2% with incomplete high school, 4% with complete high school, 11% with incomplete graduate studies, 29% graduate and 54% postgraduate. When comparing this group with the group that noticed the strategy, the only statistical difference occurred for education (*p* = 0.047—U-Mann-Whitney test).

Table 3 shows data related to the changes in food choice according to demographic variables and questions presented to consumers as well as the Odds Ratio and the *p*-value of the analysis.

## 4. Discussion

Eating is also a source of pleasure and comfort and reflects our personal and cultural characteristics, social status and relationships. Therefore, food choice is highly complex and determined by interrelating factors such as availability, liking, quality, information, and comprehension of the report [4]. Since studies [19,23,24,25] have shown that the use of traffic lights facilitates the understanding of labeling information and this comprehension is the most important strategy to induce healthier food choices, we proposed a method composed by dish nameplates. This method contains the rating scale (Figure 1) in association with the information about the composition of a healthier plate (Figure 2) to help consumers to make healthier plates.

We used the BFG [30,31] and My plate [32] to compose the groups of foods that should be consumed at lunch by Brazilians. With the information of portion, energetic value and that, lunch in Brazil corresponds to about 800 kcal; it was possible for consumers to make their choices.

It is important to highlight that vegetables are essential at lunch and they have a low ED. Therefore, this component represents a high percentage of the plate’s visual, although it corresponds to only 20% of the plate. In Brazil, restaurants offer fruits at lunch, as a kind of dessert and their portions are not big at lunch. We usually eat fruits at breakfast, small meals (like snacks) and supper after dinner. At Brazilian lunch meal, we recommend one portion of fruit, based on a percentage of TEV.

Healthy oils, nuts and/or seeds are very important components of lunch regarding the contribution for healthy fats. Although they have high ED, we recommend their consumption in a small portion a day (15% of TEV) to compose a healthier plate. They are at the center of the plate figure because they are a complement to other preparations at lunch meal.

As mentioned before, in Brazil, the consumption of white rice and beans in stock is a habit. White rice and beans in stock are the most frequently consumed food combination by the Brazilian population, with a mean per capita intake of 343.2 g per day [34]. Based on a combination of amino acids, we recommend the proportion of two parts of rice to one part of beans. We distributed the TEV of this combination as 2/3 for rice (20% of TEV of healthy grains) and 1/3 for beans (10% of TEV). It is important to highlight that in Brazil we consider protein/main dishes those protein preparation offered on the menu, usually of animal origin (except on vegetarian menus) and decisive for the selection of other items [41,42]. Therefore, we included eggs, meat, and dairy products to compose the protein group on the lunch plate.

According to Zandonadi et al. [38], the protein group represents about 21% of the plate offered to consumers from 37 Brazilians food services. It is evident the significant consumption of white rice and beans in stock (corresponding to about 52% of plate) [38], probably due to the type of food service (popular restaurants which attend low income population), that in Brazil uses white rice and beans in stock (consumption habit) as an energy source, in high portions to reduce the cost of meals.

### Food Service Application of the Food Rating Scale

In Brazil, national surveys show the increasing percentage of calories consumed out of home going from 24% in 2002 to 31% in 2009 [34] and studies indicated an association between eating out and low Brazilian dietary quality [43,44]. Understanding the cultural practices of eating out depends on analyses of what foods tend to converge within a diet pattern or within a meal since it is the entire meal and not one dish item that should classify the plate as healthy or unhealthy [43,44]. Therefore, we recommend that the food service unit adjusts its menu and TPFs to guide consumers to healthy choices at lunch, using the rating scale (Table 1 and Figure 1) and BHLP (Figure 2) to contribute for that.

Although some preparations could be changed before the strategy implementation, others such as some regional preparations [45] could not be changed because they would mischaracterize them. Therefore, if the restaurant maintains on the menu, their nameplate would be an alert to consumers that they should eat these preparations less frequently or in small portions, choosing another dish of the same group to complete the plate. Since Brazilians usually eat 500 g [31,34] of food on their lunch, by only reducing portions sizes, we would reduce the amount of food on their plates. Studies [4,46,47] have shown that portion size is a significant determinant of food intake; therefore, we cannot merely guide the reduction of portions of the dish, but we should guide consumers to compose their lunch meal in a balanced and healthy way, regarding nutrients, habits and the other factors related to feeding. Therefore, the strategy adopted needs to encourage the food service to make changes in dish preparation (those that are possible) to reduce ED and SC so that preparations could receive a better classification. It is essential to change the way the restaurant prepares the dish, so that, with the same determined portion, we can offer the crucial nutrients planned for the meal.

Regarding SC in 100 g of food, we mostly classified salad sauces as high. Despite their ED, classified as medium ED, we classified preparations with red cards. It is important to highlight that we do not recommend the use of salad sauce because they usually present high sodium content. Instead, we recommend the use of healthy oils (15% of TEV), such as olive oil, to complement the salad without the addition of salty ingredients. Restaurant foods can be an important source of sodium in the diet. Restaurant foods can contribute nearly to 25% of the sodium consumed [11,48]. A study proposed Strategies to Encourage Sodium Reduction in Restaurants. Some of these strategies are training the restaurant employees to understand why the reduction of SC is fundamental, and that consumers may desire and will purchase lower-sodium options. Therefore, they can offer healthier options to consumers to choose; incentivizing sodium reduction on foods and providing nutrition information to lead the consumers to select the best choices for them. The need for efforts to make restaurants to reduce consumer sodium exposure and to generate greater consumer awareness on how much sodium is in foods consumed away from home is noteworthy. However, there is a challenge for restaurant employees which is the lack of standardized recipes and the lack of employee knowledge about the importance of the SC on consumer health [11,29]. Recipe analysis and subsequent menu-labeling require cost and time, which can be a barrier to consumer information [29]. Concerns about restaurants profitability have also been reported as barriers to change tasty menu to healthy menu (which is considered tasteless than the menu rich in sodium and fat by consumers) [11].

Recently, Food and Drug Administration [49] published its recommendation to classify the SC in foods and considered as a rule: 5% DV or less of sodium per serving is low; 20% DV or more is high. Additionally, Food and Drug Administration (FDA) set claims as 140 mg of sodium or less per serving is “low sodium”; 35 mg of sodium or less per serving is “very low sodium”. If we classify SC by the FDA rule, salad sauces would be classified as medium sodium because FDA considers the sodium in the portion. Since portions of salad sauce are small, we would classify all sauces as low sodium, but they were not when analyzing 100 g of the dish.

The *World Cancer Research Found* recommends (WCRF) as a public health strategy that lunch should be around 1.25 kcal/g for ED [50], and the Centers of Disease Control and Prevention [27] classifies the ideal ED below 1.5 kcal/g. The mean value of these two recommendations is 1.37 kcal/g, close to the obtained in this study in food service unit (1.35 ± 0.57 kcal/g). The ED found in the study of Canella [51], for lunch meals in 21 restaurants of the city of São Paulo, was 1.43 kcal/g close to the parameters of the WCRF [50] and the CDC [27].

On the other hand, a study conducted by Lipi [52], in the city of São Paulo, showed an ED of 1.94 kcal/g for the diet of factory employees, close to the results of another study [53] with an ED of 1.98 kcal/g conducted with 710 adults using 24H recall. Kant and Graubard [54] found ED of 1.92 kcal/g in their study with 13,400 consumers in New York. All these studies show higher ED than in the present study showing menus inadequacies.

Although healthy meals relate to vegetables, some salads had ingredients that raised ED and SC such as mayonnaise, dried tomato, and soy sauce, among others. Raw and cooked vegetables presented low ED and SC because the restaurant did not use other ingredients in their preparation. These vegetables received green nameplate cards. Studies suggest targeting the food environment to promote desirable food choices in testing nudges to encourage vegetable consumption [4,55]. The authors suggest that serving vegetables in a default portion size and the visual variety of vegetables could influence the vegetable intake. They suggest that these strategies influence food choices and, the behavior is not the only way. It is important to use strategies of nutritional education (as the use of active methodologies) to influence the healthier food choice based on the automatic decision-making system, as we conducted in the present study.

Some dishes served in restaurants need to be modified to reduce ED and SC since eating out is associated with overweight, obesity, and cardiovascular diseases among adults in Brazil [38,51]. De Menezes et al. [44], in São Paulo, evaluated the impact of substituting 1720 typically consumed products in the Brazilian diet for products that follow the criteria of a healthy diet. For that, they compared nutritional labels, and modified some commercial products such as tomato sauce for homemade tomato sauce. Research showed that these substitutions could reduce the intake of saturated fat by 52%, trans fat by 92%, ED by 14%, and SC by 47%, besides increasing the intake of fiber in 87%.

Regarding the influence on consumer food choice, there was a significant difference between those who changed their food choice and those who did not change their food choice when compared to the number of items that attracted the most (Table 3). Innovative intervention strategies that can effectively improve food choices, dietary intake and impact on health have an underlying assumption that people, most of the time, make conscious and reasoned food choices. Current paradigms place the burden and responsibility for all food choices on the individual, with the justification that everyone is free to make healthy choices once informed [56].

The items that attracted the most were information about the portion size of each preparation; the use of colors (red, yellow and green) as healthier or unhealthier indicator; instructions about the amount of protein portion, carbohydrate, vegetable. Three or more items significantly attracted the individuals (*p* = 0.033) influencing the change in food choice. Also, it increased by 1.7 times the chance of food choice changes to occur. It shows that the nutritional information associated with the active methodologies (a tendency in education where the protagonist is the consumers in the case of a restaurant [57,58,59]) is related to the change in food choice, as seen in the studies conducted by Jomori [60], Hwang [61], Ozdemir [62], and Hoefkens et al. [9]. Therefore, the nutrition information interventions may be more effective when using nutrition information in combination with an educational intervention [9]. The learning process occurs with consumers being co-responsible for them on formation and changes. These studies show that when the menu presents adequate nutritional information, it increases the perception of taste, quality, and healthful aspects of food, influencing consumer behavior and their understanding of the food.

Another study aimed to identify consumer preference for nutritional information of the menu. They evaluated 180 consumers (mean age: 43 years old; frequency of lunch out of home: 2.44 times a week), and they assessed if the exposure of the nutritional information would influence on healthy choices. The study showed that consumers preferred food with the data “low-fat content”, “low in calories” and the information about the number of macronutrients. They also observed that when the food service presents the nutritional information to support healthy choices, consumers tend to identify these items and tend to prefer them on behalf of the unhealthy foods [61].

Similarly, another study carried out in two food service units with 304 consumers aimed to analyze the relationship between the reading of labels of industrialized foods and the healthy choices on the restaurants that provide nutritional information on the menu. The authors concluded that consumers who usually read food labels tend to make healthy decisions in restaurants that offer nutritional information on their menu [63].

Based on the evidence, it is noteworthy that nutritional education strategies should be permanent to stimulate consumer knowledge and healthy food choices. Also, it is essential to develop and to evaluate the TPFs for all food prepared in the food service allowing modifications in the food preparations (or on the menu) to reduce the prevalence of food preparation with red and/or yellow classification. Therefore, consumers will be able to choose healthy options for a healthy menu.

It is important to highlight that our study was conducted in only one food service with consumers with a high educational level, which represents only a little portion of our population. The educational level of consumers of this study food service can be bias when analyzing results since the majority presented a high educational level and they could better understand the strategy. Therefore, further studies are necessary to test the methodology on different groups of consumers.

## 5. Conclusions

This study elaborated a methodology of nutritional education to classify food dishes served in a lunch buffet at a food service. This methodology provides nutritional information for healthier choices to consumers, and we classified foods by traffic light colors to gather consumer attention for the ED and SC of their meals. It is a more natural way to choose among many dishes, helping them to put specific food groups on their plates.

Regarding the evaluation of the active methodology, the number of items with nutritional information exposed to consumers in the buffet showed to be a consistent indicator of the strategy. The nutritional information may influence consumer food choice. Almost half of the consumers that returned the questionnaires reported changes in food choice by using the nameplates. It is important to highlight that up to 2 items drew the attention of consumers, but almost 25% of the consumers that changed their eating behavior noticed more than three items presented on the nameplate.

Nutritional education strategies are essential in food service, especially for overweight populations. People are eating out of their homes more, but they do not know the quality of what they are eating. Trustworthy and accessible information is essential for healthy choices. However, a limiting factor of this study is that nutritional education cannot be the only strategy for behavioral change. This change presents different types of determinants such as environmental, physical, individual and social. Consumers can change temporarily, but not in the long term. Other strategies need to be developed so that consumers can change at home or when buying items in the supermarket or eating out in different spaces.

## Figures and Tables

**Table 1:**
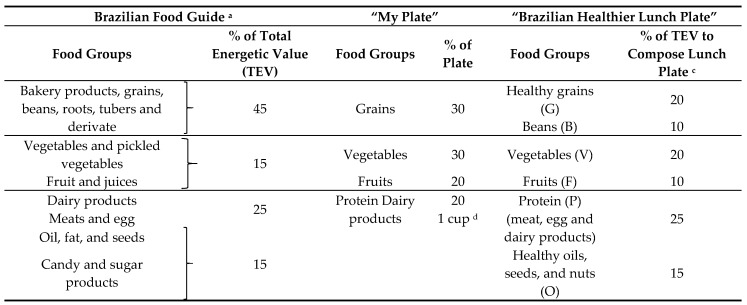
Food Groups distribution according to Brazilian Food Guide ^a^ and “My plate” and Recommendations for the “Brazilian healthier lunch plate”.

^a^ Brazilian Food Guide ^13^; ^b^ “My plate” ^15^; ^c^ Average of energy value for lunch as 800 kcal in BFG classification. ^d^ “My plate” does not present the consumption recommendation of dairy products in percentage just advice consumers to have at least 1 cup (250 mL) of dairy products.

**Table 2:** Average energy density, sodium content and portion weight of the preparations of a food service unit.

Menu Types of Preparations	Energy Density (kcal/g)	Sodium Content (mg/100 g)	Portion (g)
	Mean ± SD	Mean ± SD	Mean
Salad sauces *	1.8 ± 0.45	876 ± 806	22
Fruits	0.4 ± 0.09	4 ± 4	75
Proteins	1.6 ± 0.56	440 ± 261	69
Grains	1.6 ± 0.34	308 ± 272	103
Beans	2.0 ± 1.22	331 ± 287	69
Cooked vegetables	1.4 ± 0.89	282 ± 264	63
Raw vegetables	0.8 ± 0.55	37 ± 45	102

* These salad sauces are used to season raw vegetables and include oils, seeds and/or nuts and sauces based on mayonnaise, mustard, catchup, shoyu and other.

**Table 3:** Evaluation of the methodology by consumers that observed the nutritional education strategy and changes in food choices at a food service unit.

		Consumers Changed the Food Choice				OR ^#^	CI (OR) ^##^	*p*-Value
		Yes		No				
		N	%	N	%			
Sex	Female	139	53	113	52%	1.06	0.73–1.54	0.783
	Male	121	47%	104	48%			
	Total	260		217				
Age range	up to 35 years	118	55%	93	50%	1.2	0.79–1.82	0.369
	36 years and over	98	45%	93	50%			
	Total	216		186				
Education	Graduate	131	51%	91	43%	1.37	0.94–2.01	0.096
	Postgraduate	128	49%	122	57%			
	Total	259		213				
Marital status	Married/with mate-partner	154	61%	143	67%	0.77	0.51–1.15	0.175
	Single	97	39%	69	33%			
	Total	251		212				
Children	Has children	120	48%	108	52%	0.86	0.59–1.27	0.454
	Does not have children	130	52%	101	48%			
	Total	250		209				
Weekly frequency that have lunch on food service	Up to 2 times a week	45	18%	23	11%	1.76	1.00–3.13	0.051
	3 to 5 times a week	212	82%	191	89%			
	Total	257		214				
Number of items that called the attention	3 items or more	58	22%	31	14%	1.70	1.05-2.75	0.033 *
	Up to 2 items	202	78%	184	86%			
	Total	260		215				
Clarity of methodology	I did not understand anything	3	1.2%	2	0.9%	1.26	0.21–7.61	0.582
	I understand	256	98.8%	215	99.1%			
	Total	259		217				
What has changed in the meal after the methodology?	1 item	188	75%	9	100%	0	0–1.82	0.119
	2 items or more	63	25%	0	0%			
	Total	251		9				

^#^ OR: Odds Ratio; ^##^ CI: Confidence Interval; *: *p* ≤ 0.05.

## References

[1] Gallian D.M.C. (2007). A desumanização do comer. Estud. Avançados.

[2] Naska A., Katsoulis M., Orfanos P., Lachat C., Gedrich K., Rodrigues S.S.P., Freisling H., Kolsteren P., Engeset D., Lopes C. (2015). HECTOR Consortium Eating out is different from eating at home among individuals who occasionally eat out. A cross-sectional study among middle-aged adults from eleven European countries British Journal of Nutrition. Br. J. Nutr..

[3] Wu H.W. (2015). Unsavory choices: The high sodium density of U.S. chain restaurant foods. J. Food Compos. Anal..

[4] Friis R., Skov L.R., Olsen A., Appleton K.M., Saulais L., Dinnella C., Hartwell H., Depezay L., Monteleone E., Giboreau A. (2017). Comparison of three nudge interventions (priming, default option, and perceived variety) to promote vegetable consumption in a self-service buffet setting. PLoS ONE.

[5] Bezerra I.N., Sichieri R. (2010). Características e gastos com alimentação fora do domicílio no Brasil. Rev. Saude Publica.

[6] Lachat C., Nago E., Verstraeten R., Roberfroid D., Van Camp J., Kolsteren P. (2012). Eating out of home and its association with dietary intake: A systematic review of the evidence. Obes. Rev..

[7] Popkin B.M., Adair L.S., Ng S.W. (2012). Global nutrition transition and the pandemic of obesity in developing countries. Nutr. Rev..

[8] Wu H.W., Sturm R. (2013). What’s on the menu? A review of the energy and nutritional content of US chain restaurant menus. Public Health Nutr..

[9] Hoefkens C., Pieniak Z., Van Camp J., Verbeke W. (2012). Explaining the effects of a point-of-purchase nutrition-information intervention in university canteens: A structural equation modelling analysis. Int. J. Behav. Nutr. Phys. Act..

[10] Cunha D.B., Verly Junior E., Paravidino V.B., Araújo M.C., Mediano M.F.F., Sgambato M.R., de Souza B.D.S.N., Marques E.S., Baltar V.T., de Oliveira A.S.D. (2017). Design of a school randomized trial for nudging students towards healthy diet and physical activity to prevent obesity: PAAPAS Nudge study protocol. Medicine.

[11] Levings J.L., Gunn J.P. (2014). From Menu to Mouth: Opportunities for Sodium Reduction in Restaurants. Prev. Chronic Dis..

[12] Cranage D.A., Conklin M.T., Lambert C.U. (2004). Effect of Nutrition Information in Perceptions of Food Quality, Consumption Behavior and Purchase Intentions. J. Foodserv. Bus. Res..

[13] Deon B.C., Medeiros L.B., Lúcia de Freitas Saccol A., Hecktheuer L.H., Saccol S., Naissinger M. (2014). Good food preparation practices in households: A review. Trends Food Sci. Technol..

[14] De Carvalho A.P., De Oliveira V.B., Dos Santos L.C. (2007). Hábitos alimentares e práticas de educação nutricional:atenção a crianças de uma escola municipal de BeloHorizonte, Minas Gerais Food habits and nutritional education practices: Warning for children of a municipal school of Belo Horizonte, Minas Gerais. Pediatria (Santiago).

[15] Botelho R.B.A., Avena F., Veras M., Zandonadi R.P. (2014). Nutritional adequacy of meals offered and consumed by soldiers of the Brazilian Army. Rev. Nutr..

[16] Sanin V., Pfetsch V., Koenig W. (2017). Dyslipidemias and Cardiovascular Prevention: Tailoring Treatment According to Lipid Phenotype. Curr. Cardiol. Rep..

[17] De Oliveira C., Marmot M.G., Demakakos P., Vaz de Melo Mambrini J., Peixoto S.V., Lima-Costa M.F. (2016). Mortality risk attributable to smoking, hypertension and diabetes among English and Brazilian older adults (The ELSA and Bambui cohort ageing studies). Eur. J. Public Health.

[18] Fernandes A.C., Oliveira R.C., Proença R.P.C., Curioni C.C., Rodrigues V.M., Fiates G.M.R. (2016). Influence of menu labeling on food choices in real-life settings: A systematic review. Nutr. Rev..

[19] Sinclair S.E., Cooper M., Mansfield E.D. (2014). The influence of menu labeling on calories selected or consumed: A systematic review and meta-analysis. J. Acad. Nutr. Diet..

[20] Scholderer J., Brunsø K., Bredahl L., Grunert K.G. (2004). Cross-cultural validity of the food-related lifestyles instrument (FRL) within Western Europe. Appetite.

[21] Almiron-Roig E., Solis-Trapala I., Dodd J., Jebb S.A. (2013). Estimating food portions. Influence of unit number, meal type and energy density. Appetite.

[22] Arno A., Thomas S. (2016). The efficacy of nudge theory strategies in influencing adult dietary behaviour: A systematic review and meta-analysis. BMC Public Health.

[23] Borgmeier I., Westenhoefer J. (2009). Impact of different food label formats on healthiness evaluation and food choice of consumers: A randomized-controlled study. BMC Public Health.

[24] Balcombe K., Fraser I., Di Falco S. (2010). Traffic lights and food choice: A choice experiment examining the relationship between nutritional food labels and price. Food Policy.

[25] Dodds P., Wolfenden L., Chapman K., Wellard L., Hughes C., Wiggers J. (2014). The effect of energy and traffic light labelling on parent and child fast food selection: A randomised controlled trial. Appetite.

[26] Pereira M.G. (1995). Epidemiologia: Teoria e Pratica.

[27] Can Eating Fruits and Vegetables Help People to Manage Their Weight?. https://www.cdc.gov/nccdphp/dnpa/nutrition/pdf/rtp_practitioner_10_07.pdf.

[28] Front of Pack Nutritional Signpost Labelling Technical Guidance. https://www.foodwatch.org/fileadmin/Themen/Ampelkennzeichnung/guidance_ampel_issue_1_januar_2007.pdf.

[29] De Akutsu R.C., Botelho R.A., Camargo E.B., Sávio K.E.O., Araújo W.C. (2005). A ficha técnica de preparação como instrumento de qualidade na produção de refeições. Rev. Nutr..

[30] GUIA ALIMENTAR PARA A POPULAÇÃO BRASILEIRA Promovendo a Alimentação Saudável. http://bvsms.saude.gov.br/bvs/publicacoes/guia_alimentar_populacao_brasileira_2008.pdf.

[31] Brasil Guia Alimentar Para a População Brasileira Promovendo a Alimentação Saudável. http://dab.saude.gov.br/portaldab/biblioteca.php?conteudo=publicacoes/guia_alimentar2014.

[32] USDA My Plate. https://www.choosemyplate.gov/.

[33] Barbosa L. (2007). Feijão com arroz e arroz com feijão: OBrasil no prato dos Brasileiros. Horiz. Antropol..

[34] Instituto Brasileiro de Geografia e Estatística (2011). Pesquisa de Orçamentos Familiares: Análise do Consumo Alimentar Pessoal No Brasil.

[35] Peyerl F.F., De Matos K.H.O. (2012). Avaliação da legislação aplicada a rotulagem de alimentos embalados no Brasil e na Nova Zelândia. Rev. E-Tech Tecnol. Compet. Ind..

[36] Araújo H.M.C., Araújo W.M.C., Botelho R.B.A., Zandonadi R.P. (2010). Doença celíaca, hábitos e práticas alimentares e qualidade de vida. Rev. Nutr..

[37] Eid da Rosa A. (2013). Substitutos de Leite Condensado a Partir de Extratos Vegetais.

[38] Zandonadi R.P., Botelho R.B.A., Ginani V.C., De Cássia R., Akutsu C.A., Eleonora De Oliveira Savio K., Araújo W.M.C. (2014). Sodium and health: New proposal of distribution for major meals. Health.

[39] Mangabeira Júnior A.S., Sávio K.E.O., Pineli L.D.L.D.O., Akutsu R.D.C.C., Botelho R.B.A. (2017). Acceptability of Reduced-Fat and Fried-Food-Free Menu in Self-Service Restaurant. J. Culin. Sci. Technol..

[40] Tabela Brasileira de Composição de Alimentos, Núcleo de Estudos e Pesquisas em Alimentação (2011). TACO.

[41] Domene S.M.Á. (2011). Técnica Dietética: Teoria e Aplicações.

[42] Ginani V.C., Araújo W.M.C., Botelho R.B.A., Akutsu R.C.C.A., Zandonadi R.P. (2017). What is Offered by Public Foodservices for Low Income Population in Brazil is Adequate to Health Promotion Regarding Energy Density. J. Culin. Sci. Technol..

[43] Andrade G., da Costa Louzada M., Azeredo C., Ricardo C., Martins A., Levy R. (2018). Out-of-Home Food Consumers in Brazil: What do They Eat?. Nutrients.

[44] De Menezes E.W., Lopes T.D.V.C., Mazzini E.R., Dan M.C.T., Godoy C., Giuntini E.B. (2013). Application of Choices criteria in Brazil: Impact on nutrient intake and adequacy of food products in relation to compounds associated to the risk of non-transmissible chronic diseases. Food Chem..

[45] Duarte I.A.E., Botelho R.B.A., Akutsu R.D.C. (2017). Regional Food Consumption in the Northeast of Brazil by the Low-Income Population. J. Culin. Sci. Technol..

[46] Diliberti N., Bordi P.L., Conklin M.T., Roe L.S., Rolls B.J. (2004). Increased Portion Size Leads to Increased Energy Intake in a Restaurant Meal. Obes. Res..

[47] Ello-Martin J.A., Ledikwe J.H., Rolls B.J. (2005). The influence of food portion size and energy density on energy intake: Implications for weight management. Am. J. Clin. Nutr..

[48] Centers for Disease Control and Prevention (CDC) (2012). Vital signs: Food categories contributing the most to sodium consumption–United States, 2007–2008. MMWR. Morb. Mortal. Wkly. Rep..

[49] Sodium in Your Diet Use the Nutrition Facts Label and Reduce Your Intake. https://www.fda.gov/downloads/Food/IngredientsPackagingLabeling/UCM315471.pdf.

[50] WCRF, AICR (2007). Food, Nutrition, Physical Activity, and the Prevention of Cancer: A Global Perspective.

[51] Canella D.S., Bandoni D.H., Jaime P.C. (2011). Densidade energética de refeições oferecidas em empresas inscritas no programa de alimentação do Trabalhador no município de São Paulo. Rev. Nutr..

[52] Lipi M. (2008). Densidade Energética da Dieta de Trabalhadores de uma Indústria da Região Metropolitana de São Paulo. Master’s Thesis.

[53] Stella R.H. (2008). Densidade Energética: Relação com Variáveis Demográficas, de Estilo De vida, Nutricionais e Socioeconômicas em Amostra Representativa Da População Adulta do Município de São Paulo. Bachelor’s Thesis.

[54] Kant A.K., Graubard B.I. (2005). Energy density of diets reported by American adults: Association with food group intake, nutrient intake and body weight. Int. J. Obes..

[55] Hanks A.S., Just D.R., Wansink B. (2013). Smarter lunchrooms can address new school lunchroom guidelines and childhood obesity. J. Pediatr..

[56] Bucher T., Collins C., Rollo M.E., Mccaffrey T.A., De Vlieger N., Van Der Bend D., Truby H., Perez-Cueto F.J.A. (2016). Nudging consumers towards healthier choices: A systematic review of positional influences on food choice. Br. J. Nutr..

[57] Cotta R.M.M., de Mendonça É.T., Costa G.D. (2011). Da Portfólios reflexivos: Construindo competências para o trabalho no Sistema Único de Saúde. Rev. Panam. Salud Pública.

[58] Cotta R.M.M., da Costa G.D., Mendonça É.T. (2013). Portfólio reflexivo: Uma proposta de ensino e aprendizagem orientada por competências. Cien. Saude Colet..

[59] Mitre S.M., Siqueira-Batista R., Girardi-de-Mendonça J.M., de Morais-Pinto N.M., Meirelles C.D.A.B., Pinto-Porto C., Moreira T., Hoffmann L.M.A. (2008). Metodologias ativas de ensino-aprendizagem na formação profissional em saúde: Debates atuais. Cien. Saude Colet..

[60] Escolha Alimentar do Comensal de um Restaurante por Peso. https://repositorio.ufsc.br/xmlui/handle/123456789/88477.

[61] Hwang J., Lorenzen C.L. (2008). Effective nutrition labeling of restaurant menu and pricing of healthy menu. J. Foodserv..

[62] Ozdemir B., Caliskan O. (2015). Menu Design: A Review of Literature. J. Foodserv. Bus. Res..

[63] Roseman M.G., Mathe-Soulek K., Higgins J.A. (2013). Relationships among grocery nutrition label users and consumers’ attitudes and behavior toward restaurant menu labeling. Appetite.

